# Noncardiac Surgery in Patients with Left Ventricular Assist Devices: A 11-Year Institutional Experience

**DOI:** 10.21470/1678-9741-2023-0258

**Published:** 2024-04-15

**Authors:** Emel Gündüz

**Affiliations:** 1 Department of Anesthesiology and Reanimation, Faculty of Medicine, Akdeniz University, Antalya, Turkey

**Keywords:** Retrospective Studies, Heart-Assist Devices, Hospital Mortality, Heart Failure, Hospital Emergency Service

## Abstract

**Introduction:**

Limited options in the end-stage treatment of heart failure have led to increased use of left ventricular assist devices. For this reason, the rate of non-cardiac surgeries in patients with left ventricular assist devices is also increasing. Our study aims to analyze surgical rate, anesthesia management, and results by reviewing our 11-year experience with patients who underwent non-cardiac surgery receiving left ventricular assist devices support.

**Methods:**

We retrospectively evaluated 57 patients who underwent non-cardiac surgery and 67 non-cardiac surgical procedures among 274 patients who applied between January 2011 and December 2022 and underwent left ventricular assist devices implantation with end-stage heart failure.

**Results:**

Fifty (74.6%) patients with left ventricular assist devices admitted to the hospital for non-cardiac surgery were emergency interventions. The most common reasons for admission were general surgery (52.2%), driveline wound revision (22.3%), and neurological surgery (14.9%). This patient group has the highest in-hospital mortality rate (12.8%) and the highest rate of neurological surgery (8.7%). While 70% of the patients who underwent neurosurgery were taken to surgery urgently, the International Normalized Ratio values of these patients were between 3.5 and 4.5 at the time of admission to the emergency department.

**Conclusion:**

With a perioperative multidisciplinary approach, higher morbidity and mortality risks can be reduced during emergencies and major surgical procedures.

## INTRODUCTION

**Table:** 

Abbreviations, Acronyms & Symbols	
AKI	= Aortic cross-clamping
BIS	= Bispectral index
DM	= Diabetes mellitus
GA	= General anesthesia
GI	= Gastrointestinal
INR	= International Normalized Ratio
INTERMACS	= Interagency Registry for Mechanically Assisted Circulatory Support
LVAD	= Left ventricular assist device
NCS	= Non-cardiac surgery
RV	= Right ventricular
SD	= Standard deviation
TEE	= Transesophageal echocardiography
VATS	= Video-assisted thoracoscopic surgery

Left ventricular assist devices (LVADs) are of great importance and are frequently used in salvage, bridging to transplantation, or permanent destination treatment of patients with acute or chronic end-stage heart failure. In these patients, the primary objective is to improve symptoms, quality of life, and survival. With the increase in the survival rate of patients with LVADs, non-cardiac complications that require surgical treatment are becoming more prevalent. The rate of non-cardiac surgery (NCS) during the lifetime of these patients is 18-29%^[1,2]^. Many factors such as mechanical stress, need for anticoagulation, activation of fibrinolysis, platelet count, and function, physical characteristics of the device, blood volume changes, and pressure changes may cause bleeding. In addition, mucosal ischemia, the development of acquired von Willebrand syndrome, and arterial-venous malformation in the gastrointestinal (GI) tract are causes of hemorrhage^[3]^.

## METHODS

Among the 274 patients with end-stage heart failure who applied to Akdeniz University Faculty of Medicine’s Cardiovascular Surgery and Anesthesia Intensive Care Unit between January 2011 and December 2022, 57 patients underwent NCS, and 67 non-cardiac surgical procedures were retrospectively evaluated. Patients over the age of 18 years who underwent LVAD implantation were included in the study. Patients whose data could not be accessed from their records in the hospital system were excluded from the study. Patients older than 18 years who underwent LVAD implantation and noncardiac surgery were included in the study.

The Clinical Research Ethics Committee of Akdeniz University Faculty of Medicine approved the study protocol (approval number KAEK-28). Written informed consent was obtained from each patient. The study was carried out following the principles of the Declaration of Helsinki.

Patient’s preoperative demographic characteristics and comorbidities (hypertension, diabetes), time from LVAD implantation to NCS, presence of preoperative anticoagulation or antiplatelet agent (s), intraoperative anesthetic records, and operative notes were examined. Postoperative complications (acute renal failure, need for dialysis), transfusion, and in-hospital mortality were evaluated.

### Statistical Analysis

The statistical analyses were conducted using IBM Corp. Released 2015, IBM SPSS Statistics for Windows, version 23.0, Armonk, NY: IBM Corp. software. Descriptive statistical methods (mean, standard deviation, median, minimum, maximum, and percentage) were used when evaluating study data. The conformity of the quantitative data to the normal distribution was checked with the Shapiro-Wilk test. Student’s *t*-test was used to compare normally distributed quantitative changes. Statistical significance was accepted as *P*<0.05.

## RESULTS

In the period between January 1, 2011, and December 30, 2022, 57 of 274 patients (39 males, 18 females) who had an LVAD implanted at our facility underwent 67 NCS. The mean age of the patients was 52.2 ± 9.98 years. Demographic data is given in Table 1. The average number of days on support at the time of NCS was 65 days (range = 1-1440). Fifty (74.6%) patients were admitted for emergency non-cardiac procedures. The most common reasons for admission were general surgery (52.2%), driveline wound revision (22.3%), and neurological surgery (14.9%). A total of 36 (53.7%) patients had moderate-risk surgeries, while 31 (46.2%) had low-risk surgeries. Table 2 shows the types and numbers of NCS performed and the anesthesia technique applied. When all of the patients applied to the hospital, Coumadin® was used as anticoagulant treatment, 42 patients were using Coumadin® and aspirin, four patients were using clopidogrel in addition to Coumadin®, and two patients were using dabigatran etexilate in addition to Coumadin®. The mean preoperative International Normalized Ratio (INR) values of the patients were 1.37 ± 0.36. In 58 (86.5%) non-cardiac surgical procedures, aspirin/warfarin was discontinued five days before, and bridge treatment with heparin was started. In nine (13.4%) patients who were operated on urgently, anticoagulation had to be reversed with preoperative vitamin K and fresh frozen plasma. Platelet suspension was given to four (5.9%) patients, and prothrombin complex concentrate was given to two (2.9%) patients. Cryoprecipitate, recombinant coagulation factor VIIa, and recombinant fibrinogen were not administered to any of the patients preoperatively. The erythrocyte suspension was administered to 15 non-cardiac surgical procedures, and all were emergency operations. Arterial invasive blood pressure was used in 42 (62.6%) cases, central venous pressure monitoring was used in 21 (31.3%) cases, and transesophageal echocardiography (TEE) was used in four (5.9%) cases.

**Table 1:** Baseline patients’ characteristics and preoperative management.

Characteristics	(n=57)
Age, years (mean ± SD)	52.2 ± 9.98
Sex, n (%)	
Male	39 (68.4)
Female	18 (31.5)
Body mass index, kg/m^2^, SD	28.4 ± 4.1
Comorbidities, n (%)	
Hypertension	40 (70.1)
DM	28 (66.6)
INTERMACS	
1, n (%)	10 (17.5)
2, n (%)	15 (26.3)
3, 4, n (%)	32 (56.1)
Preoperative anticoagulation or antiplatelet agents used, n (%)	
Coumadin®	57 (100)
Coumadin® + aspirin	42 (73.6)
Aspirin	42 (73.6)
Clopidogrel	4 (5.9)
Dabigatran etexilate	2 (2.9)
Mean preoperative INR, n (%)	1.37 ± 0.36
Device type n (%)	
HeartMate 2™	9 (15.7%)
HeartMate 3™	12 (21%)
HeartWare™	36 (63.1%)
Preoperative management, all cases = 67, n (%)	
Arterial line	42 (62.6)
Central venosus catheter	21 (31.3)
Preoperative heparin anticoagulation	58 (86.5)

DM=diabetes mellitus; INR=International Normalized Ratio; INTERMACS=Interagency Registry for Mechanically Assisted Circulatory Support; SD=standard deviation

**Table 2:** Type of non-cardiac surgical procedures (n = 67).

Procedure	Number of procedures	Anesthetic technique
Abdominal surgery		GA
Cholecystectomy	4	
Splenectomy	2	
Abdominal exploration (rectus hematoma)	3	
Left hemicolectomy	1	
Neurologic surgery		
Craniotomy	10	GA
Thoracic surgery		
VATS	1	GA
Tracheostomy	4	GA
Orthopedic surgery		
Right foot amputation	1	Neuraxial
Colonoscopy	15	Sedation
Endoscopy	10	
Driveline/wound surgery	15	
Hysteroscopy	1	

GA=general anesthesia; VATS=video-assisted thoracoscopic surgery

All patients were evaluated postoperatively according to the Kidney Disease: Improving Global Outcomes (or KDIGO) criteria, and acute kidney injury (AKI) was detected in 11 patients (19.2%). Providing renal replacement therapy to all patients who developed AKI following surgery was necessary, while dialysis was necessary in six (54.5%) cases. Among the patients who developed AKI, 63% had undergone neurological surgery.

## DISCUSSION

The expansion of the use of LVAD in end-stage heart failure and the development of new-generation devices increase the applications for NCS due to the increase in survival in these patients. In our study, perioperative and postoperative risk factors, incidences, and methods used in these patients were examined using descriptive analysis in light of the literature.

LVAD implantation has been performed on 274 patients in our clinic since 2011. It is essential to approach the perioperative workflow of these patients with a multidisciplinary team during the NCS period. The cardiovascular surgeon should be aware of the patient during the application period. In addition, the non-cardiac surgeon who will operate should be informed about LVAD^[4,5]^. Anesthesiologists should know LVAD physiology and its effect. Coordination is established with preoperative cardiologists and, if necessary, with hematologists regarding anticoagulation. In major surgeries, patients’ flow, power, and pulse index should be continuously monitored with the LVAD control console. In our institution, non-cardiac surgical procedures were successfully applied to patients with LVAD by an experienced and trained team.

When patients with LVAD undergo NCS, the anesthetic goals are to maintain adequate LVAD forward flow and tissue perfusion. Intraoperative hemodynamic goals are to maintain adequate preload, afterload, coronary perfusion, right ventricular (RV) function, and heart rate. In addition, depth of anesthesia and analgesia should be provided and followed by bispectral index (BIS). Hypoxia, hypercarbia, and acidosis should be avoided, and it should be kept in mind that pump preload and pulsatility may decrease and cause hypotension in Trendelenburg and reverse Trendelenburg positions according to surgery. In the prone position, the LVAD cannula may become trapped, and the flow may decrease. TEE is used to evaluate fluid management, pump status, left ventricular volume, and RV functions in mediumand high-risk surgical procedures where significant hemodynamic changes are expected. Our study used TEE to monitor volume status and RV functions in four major neurologic surgeries^[6]^.

**Fig. 1:**
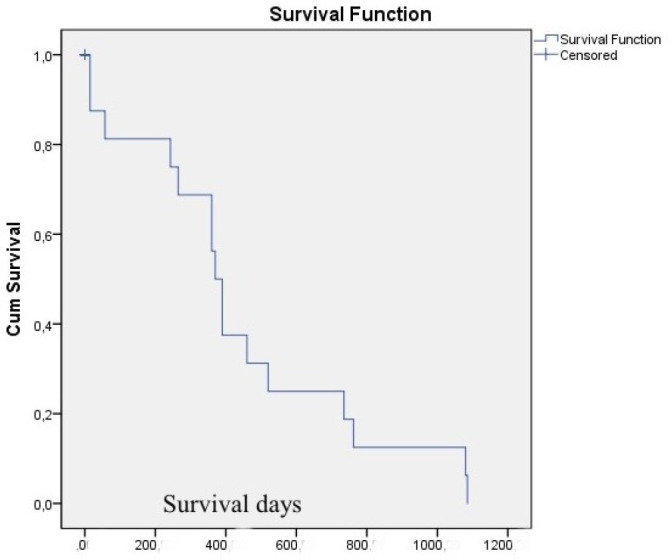
Kaplan-Meier survival curve for left ventricular assist devices undergoing non-cardiac surgery.

In a study of 3,216 cases in which the results of NCS were examined, it was reported that in-hospital mortality was 7.7%, and the highest in-hospital mortality rates were in neurosurgery (37.6%), head and neck surgery (23.5%), and thoracic surgery (11.5%). The same study showed that the need for blood transfusion was at the rate of 75% in gynecologic and 60.4% in neurological surgeries^[7]^. In our study, 57 patients who applied for 67 NCS procedures were in the group of patients who had the highest in-hospital mortality rate (12.8%) and who underwent neurological surgery (8.7%). In addition, preoperative INR was associated to mortality risk. Median survival by Kaplan-Meier analysis is shown in Figure 1. All of these patients were admitted to the emergency department with complaints such as altered consciousness, nausea, vomiting, and headache, and in 60% of them, INR values were between 3.5 and 4.5. In emergency cases, there was a need for transfusion of preoperative, intraoperative, or postoperative blood and blood products, as there was not enough time to stop oral anticoagulants and switch to bridging therapy. Fresh frozen plasma transfusion was applied to all cases; platelet was applied to 40%, cryoprecipitate to 10%, and whole blood to 60% of the cases.

In the study of Briasoulis et al.^[8]^, the rate of AKI was determined as 23% in NCS patients with LVAD. In our study, consistent with the literature, the overall rate of AKI was 19.2%, and the highest rate was 72.7% after neurosurgery.

Goudra and Singh’s study noted that non-invasive blood pressure monitoring was sufficient in 68 endoscopy procedures of 38 LVAD-supported cases^[9]^. Non-invasive hemodynamic monitoring is suitable for monitoring NCS procedures in minor cases (*e.g.*, endoscopy, colonoscopy, hysteroscopy). In our study, non-invasive blood pressure monitoring was sufficient for hemodynamic monitoring in minor surgeries (40%).

### Limitations

This study has some potential limitations. It is a single-center retrospective study, the number of cases is relatively low, and additional studies evaluating more series are needed. In addition, in recent years, TEE, cerebral oximetry, and BIS have been used during major NCS of patients with LVAD in our clinic, but we could not report it due to the insufficient number of cases.

## CONCLUSION

With the development of new-generation devices for patients with LVAD and the improvement of patient care, it is becoming increasingly common for these patients to undergo NCS.

More than half of the patients apply for emergency NCS, most commonly due to GI bleeding. Using a multidisciplinary team approach will reduce perioperative morbidity and mortality to prevent permanent sequelae in the patient, primarily because high INR values cause problems during emergency admission in the vast majority of cases undergoing neurosurgery.
